# A Natural Language Processing Framework for Structuring and Visualizing Clinical Trial Eligibility Criteria at Scale: Protocol for a Quantitative Study

**DOI:** 10.2196/86425

**Published:** 2026-05-14

**Authors:** Justin Xie, Jeet Parikh, Jessica Liu, Sameer Pandya, Guannan Gong

**Affiliations:** 1 Yale College, Yale University, New Haven, CT, United States; 2 Yale Cancer Center, Yale School of Medicine, 333 Cedar Street, New Haven, CT, 06510, United States, 1 203-200-2328; 3 Department of Laboratory Medicine, Yale School of Medicine, New Haven, CT, United States

**Keywords:** eligibility determination, clinical trials as topic, natural language processing, large language models, machine learning, data visualization, cluster analysis, breast neoplasms, lung neoplasms, gastrointestinal neoplasms

## Abstract

**Background:**

Eligibility criteria are essential to clinical trial design, guiding recruitment, and ensuring patient safety and scientific rigor. However, criteria are often lengthy, heterogeneous, and inconsistently formatted, which hinders large-scale interpretation and slows patient-trial matching. Manual review is time-consuming and error-prone. Advances in natural language processing and large language models (LLMs) offer opportunities to standardize and analyze eligibility text at scale.

**Objective:**

This study aims to develop and evaluate a scalable system that uses LLM-enabled natural language processing and unsupervised learning to identify, normalize, categorize, and visualize clinical trial eligibility criteria, with the goal of improving patient-trial matching and revealing domain-level trends.

**Methods:**

We designed a three-part pipeline: (1) representation of eligibility text using embeddings, followed by clustering to group semantically similar criteria; (2) dual-layer zero-shot LLM summarization for concept normalization, refinement, and deduplication of cluster exemplars; and (3) an interactive, web-based visualization interface to explore criteria distributions and trends by disease domain and over time. The pipeline was applied to 53,872 oncology trials (breast, lung, and gastrointestinal cancer) indexed on ClinicalTrials.gov. Outputs include cluster labels, normalized criterion summaries, and per-domain frequency profiles. Feasibility was assessed via successful end-to-end processing and inspection of face validity for cluster coherence and domain-specific patterns.

**Results:**

The system successfully processed all 53,872 trials and generated stable clusters of inclusion and exclusion concepts. The LLM summarization layers produced concise, nonredundant labels that improved the interpretability of clustered criteria. The visualization interface enabled rapid exploration of cross-trial patterns and temporal trends within breast, lung, and gastrointestinal oncology, facilitating identification of common inclusion requirements and potential barriers to enrollment. A public, open-source demonstration instance allows for interactive exploration of these clusters and summaries. Benchmarking through human validation on a random sample of eligibility criteria found the system to be 94% (470/500) accurate, reflecting its ability to consistently categorize criteria correctly in congruence with human judgment.

**Conclusions:**

A combined embeddings-clustering-LLM pipeline can standardize heterogeneous eligibility text and surface domain-level patterns at scale. This framework provides a foundation for accelerating patient-trial matching and informing future trial design. While the current implementation was evaluated on ClinicalTrials.gov oncology trials, the approach is readily generalizable to additional diseases and alternative modeling configurations.

## Introduction

### Background and Significance

Eligibility criteria are fundamental to clinical trial design, serving as the basis for patient recruitment and ensuring participant safety [1,2]. However, these criteria are often written in heterogeneous and semistructured formats across protocols, making automated interpretation and matching challenging [3,4]. Traditionally, assessing patient eligibility requires manual chart review—a time-consuming and error-prone process that contributes to screening inefficiencies and delays in trial enrollment [5,6]. To address this, automated systems have been developed to streamline patient-trial matching by extracting and standardizing relevant data from electronic health records [7-9].

Recent advancements in natural language processing (NLP) and large language models (LLMs) have created new opportunities for improving automated clinical trial matching systems and reducing recruitment timelines [10-13]. Foundational transformer-based and LLM architectures have demonstrated strong capabilities in clinical and biomedical language understanding [14-17], including medical question answering and licensing examinations [18,19], information extraction [20], and medical-oriented conversational systems [21]. LLMs have increasingly served as a backbone for automated patient-trial matching approaches [22,23].

Despite these advances, a critical gap remains: the absence of a unified framework for normalizing, summarizing, and benchmarking eligibility criteria at scale [3,10,24]. Without systematic normalization, trial designers cannot easily compare their criteria against prevailing standards, potentially leading to overly restrictive eligibility definitions that may limit accrual and reduce generalizability [2,25].

To address this need, we developed an LLM-enabled NLP and unsupervised learning system designed to summarize frequently used eligibility concepts, identify emerging trends, and support evidence-informed protocol design. In practice, this tool functions as a decision support platform for protocol optimization, enabling researchers to detect redundant or “outlier” criteria that may unnecessarily constrain the eligible patient population. Furthermore, it addresses infrastructure scalability challenges by introducing a standardized concept representation layer that facilitates cross-trial interoperability without manual reconfiguration.

Our prototype features a three-part architecture: (1) extraction and embedding of eligibility criteria, (2) unsupervised clustering with dual-layer LLM summarization, and (3) an interactive web-based exploration interface. This system provides researchers with an intuitive platform for analyzing patterns in clinical trial design and supporting scalable, model-driven enhancements to automated trial matching infrastructure.

### Study Objectives

In this study, we introduce a novel system that leverages foundational language models to identify, categorize, summarize, and visualize clinical trial eligibility criteria across disease domains. Our approach is tailored to the analysis of clinical trial protocols with the goal of improving automated patient matching and informing future trial design.

## Methods

### Overview

We developed a novel, scalable system for categorizing and visualizing clusters of clinical trial eligibility criteria with the aim of identifying recurring patterns across disease domains. The system has 3 components: unsupervised clustering of semantically similar criteria, LLM-based summarization, and an interactive visualization interface. For feasibility, we prototyped the system using clinical trial data from breast, lung, and gastrointestinal (GI) oncology. The visualization tool enables users to explore and interact with eligibility criteria clusters through features such as dynamic highlighting, panning, zooming, and time-based filtering. This allows for temporal analysis of how eligibility criteria evolve, offering new insights into clinical trial design trends across oncology subtypes.

The data used in this study were extracted from ClinicalTrials.gov via the application programming interface (API). Text embeddings were obtained through the *text-embedding-3-large* embeddings model. Clustering was completed through the k-means algorithm provided by the open-source scikit-learn library (Google Summer of Code project). Summarization was achieved through the LLM GPT-4o (OpenAI) via API. All data transmitted to external APIs consisted solely of deidentified, publicly available eligibility criteria text. No trial identifiers, patient-level data, or personal health information were included.

### Data Sources

Clinical trial data were extracted from ClinicalTrials.gov via the API for 3 oncology domains: breast cancer, lung cancer, and GI cancer. Disease-specific keywords (“breast cancer,” “lung cancer,” and “GI oncology”) were used to retrieve relevant trial information—including National Clinical Trial ID, eligibility criteria text, trial sponsor, sponsor type, and trial start date. Only trials containing complete data across all required fields were included in the analysis. All open or enrolling and closed trials were considered part of the initial dataset. The final dataset comprised 53,872 oncology trials, including 14,222 breast cancer trials, 15,624 lung cancer trials, and 24,026 GI oncology trials. These records served as the foundation for subsequent preprocessing, clustering, and analysis.

### Data Preprocessing

Given that the focus of this study was on industry eligibility criteria, analyses were restricted to industry-sponsored trials listed on ClinicalTrials.gov to maintain consistency in reporting structure and formatting. As a result, the datasets were refined to 3108 breast cancer trials, 4470 lung cancer trials, and 4539 GI oncology trials.

The second stage involved isolating inclusion criteria. Inclusion criteria were prioritized over exclusion criteria because inclusion criteria are more directly relevant to patient-trial matching. Additionally, merging both criterion types in a single analysis could lead to confusion in interpretation for end users. Thus, our system was designed to process both types independently, allowing for more interpretable clustering results.

In the final preprocessing step, we transformed the unstructured eligibility criteria text from ClinicalTrials.gov into a structured, line-by-line format suitable for downstream analysis. While most trials presented criteria as bullet points, formatting varied considerably across entries. We applied a series of regular expression filters to normalize the text—removing extraneous characters, bullet markers, line breaks, and indentation. This process yielded coherent lines of inclusion criteria, most of which reflected interpretable and meaningful content. The small subset of noisy or malformed entries was effectively neutralized through the clustering and summarization components later in the pipeline.

After preprocessing, the final datasets included 31,009 lines of inclusion criteria for breast cancer, 44,595 lines of inclusion criteria for lung cancer, and 42,555 lines of inclusion criteria for GI oncology. These were stored as structured CSV files, with each line of inclusion criteria linked to its corresponding National Clinical Trial ID and trial start date. A summary of dataset statistics is provided in Table 1.

**Table 1.** Dataset statistics in stages of data preprocessing.

	Breast cancer, n	Lung cancer, n	GI^a^ oncology, n
Total trials (raw data)	14,222	15,624	24,026
Trials after industry filter	3108	4470	4539
Lines of inclusion criteria	31,009	44,595	42,555

a GI: gastrointestinal.

### System Summary

#### Overview

**Figure 1.**
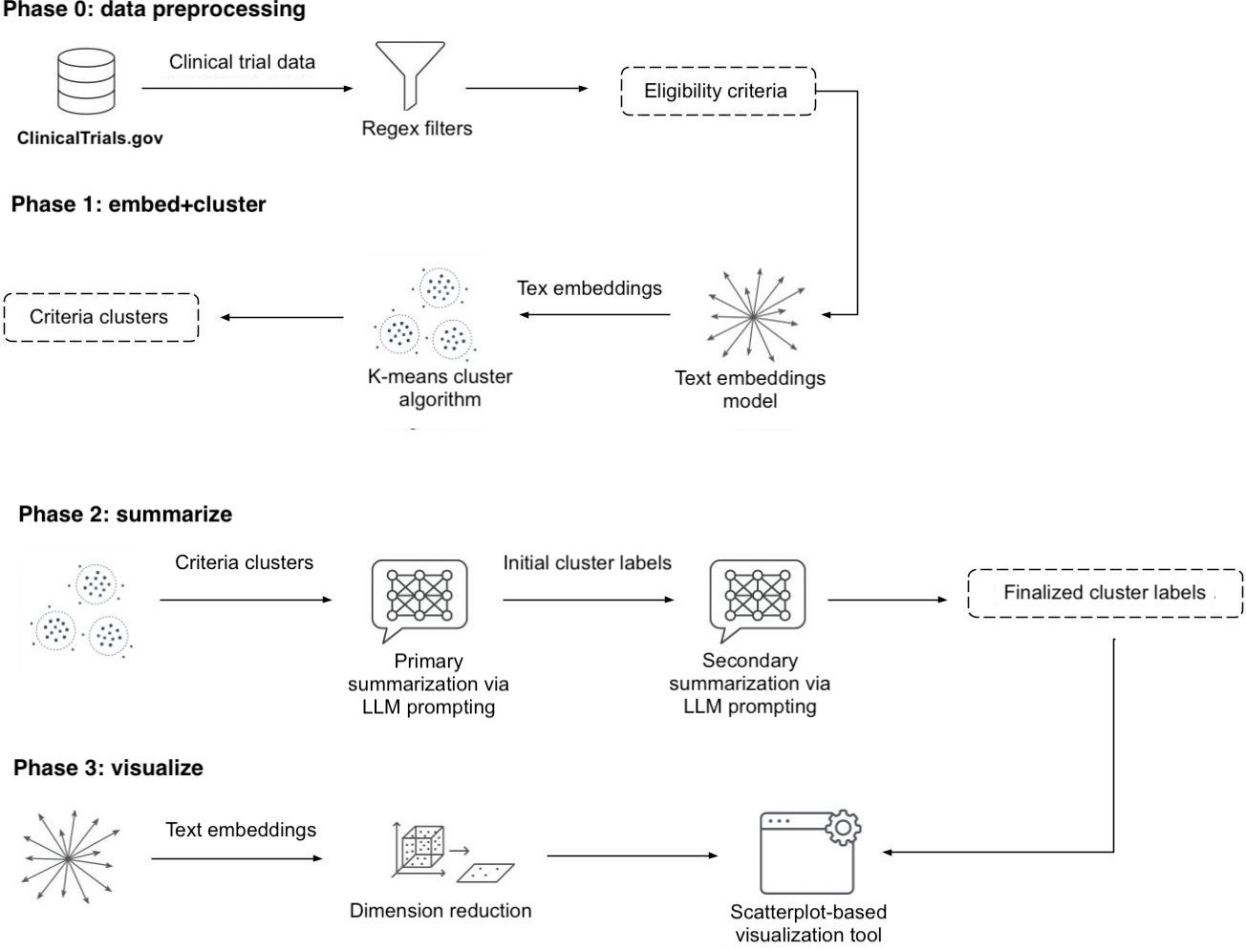
System overview: phase 1 (embed and cluster), phase 2 (dual-layer large language model [LLM] summarization), and phase 3 (visualization). Regex: regular expression.

We developed a generalizable three-phase system designed to (1) embed and cluster, (2) summarize, and (3) visualize clinical trial eligibility criteria across disease domains. The pipeline is illustrated in Figure 1. The system uses text embeddings and an unsupervised learning model to extract eligibility criteria from clinical trial records and group trials by semantic similarity. To refine the clusters, we use a generative pretrained transformer–based language model in a zero-shot setting, enabling 2 layers of summarization. The first layer summarizes the content within each cluster, whereas the second layer functions as a quality control mechanism, filtering duplicate, overly narrow, or irrelevant clusters to improve clarity and relevance. Inspired by MedViz—a platform for visualizing medical literature [26]—the web interface supports visualization of criteria clusters over time and across disease domains, with features such as time-based filtering, dynamic cluster highlighting, and smart tooltips.

#### Phase 1: Embed and Cluster

To semantically cluster and categorize the eligibility criteria data, we used OpenAI’s *text-embedding-3-large* model to generate semantically accurate, 3072D vector embeddings for each criterion. To cluster, we used a k-means clustering algorithm from scikit-learn. A cluster count of 100 was selected based on a trade-off between thematic specificity and statistical robustness. Smaller cluster counts produced overly broad groupings that obscured emerging and specialized criteria such as biomarkers (eg, *BRCA1* or *BRCA2*), whereas larger counts fragmented semantically coherent groups and resulted in clusters with insufficient density for stable summarization. We found an effective balance at a *K* value of 100 between granularity and interpretability.

To further ensure robustness, clusters containing fewer than 100 inclusion criteria were excluded to avoid instability in similarity estimation and summarization. It should be noted that the visualization tool displays small-sized clusters but no information beyond their relationship to larger, relevant clusters. For the remaining clusters, we computed average intracluster cosine similarity and applied a threshold of 0.5 to enforce semantic coherence. As unsupervised clustering does not involve class labels, traditional class imbalance mitigation was not applicable. However, to address variability in cluster sizes, the minimum threshold of 100 inclusion criteria per cluster helped ensure that all retained clusters had sufficient density for stable summarization and reduce the influence of disproportionately small groupings on downstream analysis.

Due to the unsupervised learning approach and absence of ground-truth labels, conventional performance evaluation during training was not applicable. Instead, clustering performance was assessed post hoc through a human validation benchmark whereby independent evaluators served as the ground truth for verifying cluster label assignments.

#### Phase 2: Summarize

The second phase generates summarization labels for each of the clusters. For both primary and secondary summarization, we leveraged OpenAI’s GPT-4o model and designed zero-shot prompts for cluster labeling. This model was selected based on its general language and semantic understanding. Both the *text-embedding-3-large* and GPT-4o models were used as off-the-shelf, pretrained models without additional fine-tuning. Given that GPT-4o demonstrates strong zero-shot generalization across language tasks and that no domain-specific labeled training data were available, fine-tuning was deemed unnecessary for the summarization objectives of this study.

**Figure 2.**
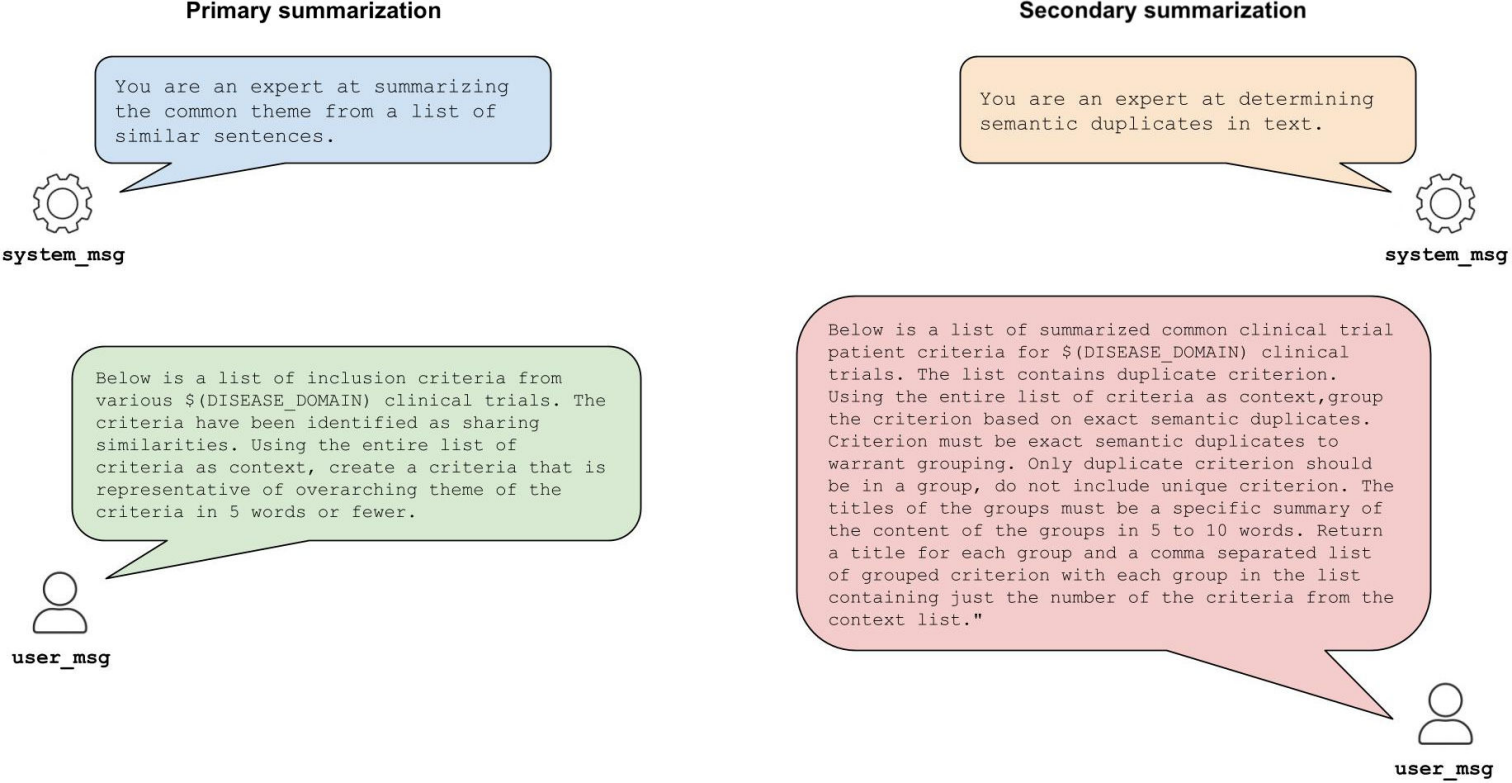
Prompting schema for primary and secondary summarization (example prompts; brief 5-word labels to minimize clutter).

In both primary and secondary summarization, a temperature of 0 and top_p of 0.1 were used to minimize variability in LLM responses and control response quality. The specifics of the prompting can be found in Figure 2.

To generate preliminary labels for each cluster, we provided 100 inclusion criteria that were randomly selected from each cluster along with a system and user prompt to the LLM. The LLM was prompted to generate a set of short, 5-word labels that provided a general summary of the specific criteria for each cluster. The short labels minimized visual clutter in the visualization application. The prompts could be altered to achieve varying levels of detail.

As primary summarization created some labels with a high degree of overlap, we used a similar prompting approach for secondary summarization whereby the LLM examined labels from primary summarization and identified semantically similar clusters. The merged clusters were given new 5-word labels by the model. The final cluster set included the merged clusters and clusters from the primary summarization that did not require reorganization.

#### Phase 3: Visualize

**Figure 3.**
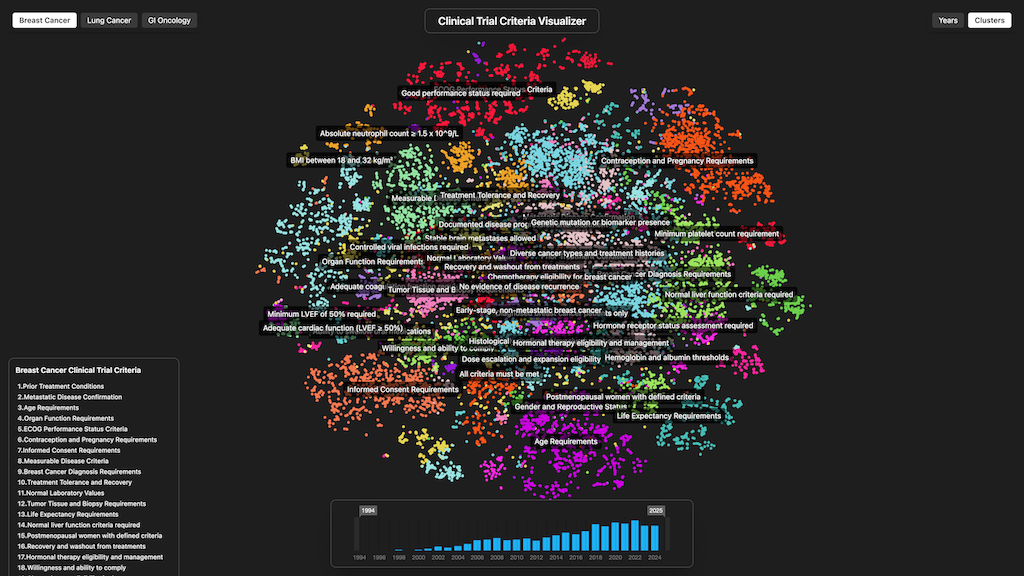
Interactive visualization: domain faceting, label hover highlighting, and dynamic cluster display.

To prepare the data for visualization, we performed a 2D reduction on all inclusion criteria embeddings using t-distributed stochastic neighbor embedding via scikit-learn. The resulting 2D coordinates were plotted on a scatterplot, with cluster label positions calculated by averaging the x and y coordinates of all criteria within each cluster. An interactive web application was developed using Three.js and React to display cluster data stratified by time and disease type. Figures3  4 provide screenshots of the visualization application. Features of the interactive tool are presented in detail in the Results section.

**Figure 4.**
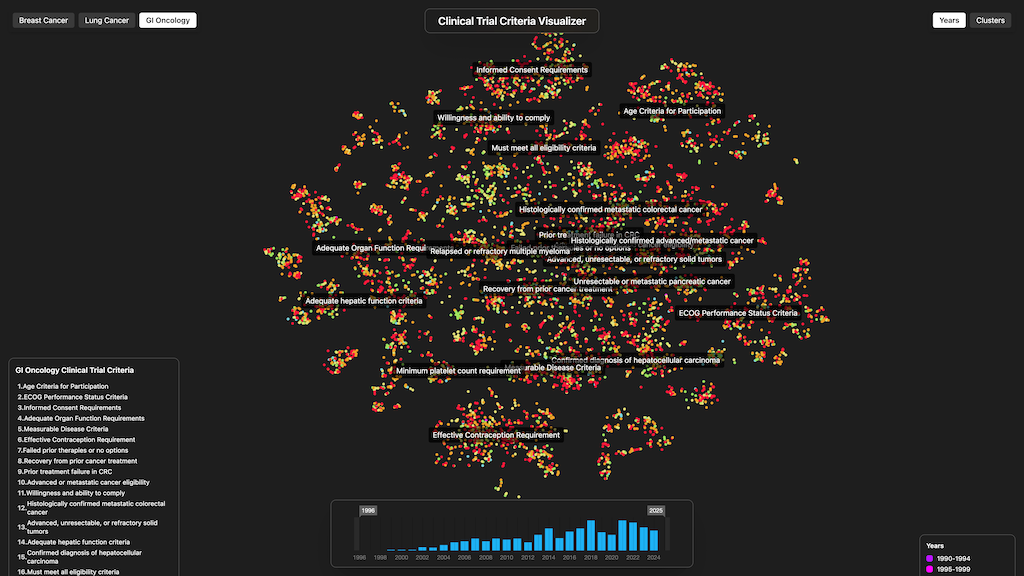
Temporal view: trial start date histogram and time window filtering.

### Ethical Considerations

This study used only publicly available, deidentified clinical trial eligibility criteria data obtained from ClinicalTrials.gov. Therefore, the study did not require institutional review board approval or informed consent.

## Results

### Overview

The culmination of our novel design and 3-phase system is a visualization tool that aims to provide users and researchers with an interactive method through which they can explore details of eligibility criteria for varying disease domains. Trends such as the distribution and evolution of criteria over time and the most common criteria can be observed using our tool. Currently, the tool allows users to interact with industry clinical trial inclusion criteria data for breast cancer, lung cancer, and GI oncology from ClinicalTrials.gov. However, following the designed pipeline of data extraction, preprocessing, clustering, summarization, and visualization, the tool is easily scalable to a wide array of disease domains. As of April 2026, the tool provides 3 features.

### Benchmarking Through Human Validation

A benchmark of the framework’s clustering accuracy was conducted using human validation, with evaluators serving as the ground truth for assessing clustering and labeling performance. A simple random sample of 500 eligibility criteria, along with the complete list of potential cluster labels, was provided to the evaluators. For each criterion, 2 independent evaluators determined whether the assigned cluster label was appropriate. Of the 500 sampled criteria, evaluators deemed 470 (94%) to have the appropriate cluster label assignments. Detailed evaluation results are presented in Table 2.

**Table 2.** Human validation benchmark results.

	Total labels evaluated, n	LLM^a^ correctly labeled samples, n	LLM incorrectly labeled samples, n	Accuracy (%)
Human evaluator 1	250	239	11	95.6
Human evaluator 2	250	231	19	92.4
Total	500	470	30	94

a LLM: large language model.

### Feature 1: Criteria Clusters

A ranking of the most common eligibility criteria clusters is displayed within the tool; see Table 3 for reference. Users can select a criteria cluster to highlight it alongside its corresponding label. The tool also includes an interactive hover feature that displays the quantity and distribution of eligibility criteria within each cluster.

**Table 3.** Most common criteria clusters summarized.

Cluster rank	Breast cancer	Lung cancer	GI^a^ oncology
1	Prior treatment conditions	ECOG^b^ performance status requirements	Age criteria for participation
2	Metastatic disease confirmation	Measurable disease criteria	ECOG performance status criteria
3	Age requirements	Effective contraception and pregnancy prevention	Informed consent requirements
4	Organ function requirements	Age requirements for participation	Adequate organ function requirements
5	ECOG performance status criteria	Prior treatment and therapy requirements	Measurable disease criteria
6	Contraception and pregnancy requirements	Minimum life expectancy criteria	Effective contraception requirement
7	Informed consent requirements	Informed consent requirements	Failed prior therapies or no options
8	Measurable disease criteria	Advanced, untreated, non–small cell lung cancer	Recovery from prior cancer treatment
9	Breast cancer diagnosis requirements	Histologically confirmed advanced non–small cell lung cancer	Prior treatment failure in CRC^c^
10	Treatment tolerance and recovery	Advanced, unresectable, or metastatic tumors	Advanced or metastatic cancer eligibility
11	Normal laboratory values	Advanced solid tumors refractory to treatment	Willingness and ability to comply
12	Tumor tissue and biopsy requirements	Performance status ≤2; life expectancy	Histologically confirmed metastatic colorectal cancer
13	Life expectancy requirements	EGFR^d^ mutation treatment failure criteria	Advanced, unresectable, or refractory solid tumors
14	Normal liver function criteria required	Negative pregnancy test and contraception	Adequate hepatic function criteria
15	Postmenopausal women with defined criteria	Tumor tissue availability and consent	Confirmed diagnosis of hepatocellular carcinoma

a GI: gastrointestinal.

b ECOG: Eastern Cooperative Oncology Group.

c CRC: colorectal cancer.

d EGFR: epidermal growth factor receptor.

### Feature 2: Interaction

The tool provides users with abilities such as zooming and panning to better interact with various areas of the scatterplot. A fixed scatter point size feature maintains point size regardless of zoom level. This allows for access to specific groups of data points and for investigation of the minutiae of criteria distribution. Another feature is label hovering. When a user hovers over the label of a cluster, all criteria within that cluster are highlighted through animation, enabling visualization of distribution and size of different inclusion criteria clusters.

### Feature 3: Time

Our tool allows users to filter displayed criteria over time. Criteria are organized by corresponding trial start date and plotted in colors corresponding to specific 5-year spans. Additionally, an interactive histogram allows users to adjust the trial start date window to filter for criteria within selected years.

## Discussion

### Anticipated Findings

In this paper, we propose a novel, generalizable pipeline for analyzing clinical trial eligibility criteria. Our system consists of 3 components—semantic clustering, 2-stage summarization via LLMs, and an interactive visualization tool—designed to extract, categorize, and present common inclusion criteria across disease domains. This approach enables scalable analysis of eligibility criteria and provides a foundation for improving automated patient-trial matching systems.

To demonstrate the feasibility of our approach, we developed a prototype site for analysis of breast cancer, lung cancer, and GI oncology trial criteria. As standardization and summarization of eligibility criteria are critical to automated patient matching systems, the proposed interactive tool may contribute to both the advancement of automated patient matching systems and the design of future clinical trials by providing valuable insights into important criteria trends. For example, the tool can be used to analyze the coverage of an automated clinical trial patient matching system, identifying the strengths and weaknesses of the system in selecting patients based on criterion areas. This has implications for patient health outcomes: overly restrictive or inconsistently applied eligibility criteria are a documented barrier to trial enrollment, particularly among underrepresented populations [27-29]. By surfacing criteria trends across thousands of trials, the tools may equip trial designers with the evidence needed to identify criteria that may unnecessarily exclude eligible patients, streamline redundant requirements, and potentially broaden access to treatments. For clinicians and trial coordinators, the tool provides a reference for benchmarking a given trial’s eligibility structure against established patterns in the field, supporting more informed and equitable protocol design.

First, our preliminary design leveraged data from ClinicalTrials.gov, focusing on a subset of industry-sponsored trials to ensure data consistency and relevance to real-world implementation. To maintain feasibility and depth of analysis, the prototype was scoped to the solid tumor domain, which represents a large and diverse set of oncology trials with substantial clinical and research interest. In addition, the clustering and summarization algorithms are far from perfect, sometimes leading to extraneous or out-of-scope clusters, labels, and results. Errors in cluster assignments or summarization labels could propagate to downstream applications, including automated patient-trial matching systems, by misrepresenting eligibility criteria categories; this risk is mitigated by the 2-stage filtration process, cosine similarity thresholding, and the human validation benchmark described in the Results section. This tool is meant to serve as a first step toward improving the coverage and capabilities of automated clinical trial patient matching systems, with the longer-term goal of reducing the time between patient identification and trial enrollment.

To expand application of the tool beyond solid tumor disease domains, future work should explore trials beyond the 3 oncology domains highlighted in this paper, as well as a wider array of foundational clustering algorithms and LLMs. Improvements in the capabilities of our designed system are also among potential future studies. For example, this may include expanding the tool beyond solid tumor oncology, expanding the trial database to additional registries and disease areas, benchmarking various clustering algorithms (eg, k-means and density-based spatial clustering of applications with noise) to improve grouping criteria clusters, and expanding the visualization dashboard with suites of filters and tools so researchers can more effectively investigate criteria coverage. These targeted enhancements will increase both the breadth and utility of the proposed system.

### Conclusions

This study demonstrates the feasibility and utility of an unsupervised, LLM-driven pipeline for structuring and visualizing clinical trial eligibility criteria at scale. By applying text embedding, clustering, and zero-shot summarization techniques, we were able to organize unstructured eligibility texts into interpretable clusters and generate domain-specific insights through an interactive visualization tool. Our findings reveal recurring patterns across disease domains—such as consistent emphasis on performance status, organ function, and prior treatment history—as well as domain-specific nuances such as differential emphasis on pregnancy prevention in breast cancer trials or molecular diagnostics in lung cancer. These patterns not only reflect current clinical practice but also point to areas where eligibility requirements could be streamlined or standardized to enhance patient access and diversity in trials.

The ability to surface and compare common eligibility themes across thousands of trials allows for more informed, data-driven protocol design, offering trial sponsors, researchers, and regulatory bodies a pathway toward reducing unnecessary complexity, improving feasibility, and minimizing barriers to enrollment. By embedding eligibility criteria into a structured, computable format, our framework also paves the way for integration with electronic health records and trial matching platforms, enhancing automation and reducing the manual burden in the recruitment process.

Although our prototype was limited to solid tumor oncology and used a subset of industry-sponsored trials from ClinicalTrials.gov, the methodology is readily generalizable. Future work will expand to additional disease areas, incorporate real-world data validation, and further optimize the NLP pipeline for deployment in clinical decision support systems. Ultimately, this framework lays the groundwork for a scalable, generalizable, and open-source infrastructure that can support more inclusive, efficient, and intelligent clinical trial design and matching.

## Abbreviations

API: application programming interface
GI: gastrointestinal
LLM: large language model
NLP: natural language processing
